# Early-Stage Oral Tongue Squamous Cell Carcinoma and a Positive Sentinel Lymph Node Biopsy: Description of a Prognostic Correlation between Pre-Treatment Inflammatory Biomarkers, the Depth of Invasion and the Worst Pattern of Invasion

**DOI:** 10.3390/jpm12111931

**Published:** 2022-11-19

**Authors:** Giovanni Salzano, Giulia Togo, Francesco Maffia, Luigi Angelo Vaira, Fabio Maglitto, Umberto Committeri, Roberta Fusco, Maria Grazia Maglione, Riccardo Nocini, Pietro De Luca, Agostino Guida, Arianna Di Stadio, Gerardo Ferrara, Luigi Califano, Franco Ionna

**Affiliations:** 1 Maxillo-Facial and ENT Surgery Unit, INT—IRCCS “Fondazione G. Pascale”, 80131 Naples, Italy; 2 Maxillofacial Surgery Unit, Department of Neurosciences, Reproductive and Odontostomatological Sciences, University Federico II, 80131 Naples, Italy; 3 Maxillofacial Surgery Operative Unit, Department of Medicine, Surgery and Pharmacology, University of Sassari, 07100 Sassari, Italy; 4 Biomedical Science Department, PhD School of Biomedical Science, University of Sassari, 07100 Sassari, Italy; 5 Oncology Medical and Research Development Division, Igea SpA, 80131 Naples, Italy; 6 Unit of Otolaryngology, Head and Neck Department, University of Verona, 37134 Verona, Italy; 7 Department of Medicine, Surgery and Dentistry, “Scuola Medica Salernitana”, University of Salerno, 84081 Baronissi, Italy; 8 U.O.C. Odontostomatologia, AORN A. Cardarelli Hospital, 80131 Naples, Italy; 9 Otolaryngology Department, University of Catania, 95123 Catania, Italy; 10 Department of Pathology, INT—IRCCS “Fondazione G. Pascale”, 80131 Naples, Italy

**Keywords:** tongue carcinoma, depth of invasion, DOI, worst pattern of invasion, WPOI, sentinel lymph node biopsy, neutrophil-to-lymphocyte ratio, NLR, platelet-to-lymphocyte ratio, maxillo-facial surgery

## Abstract

The aim of this study was to investigate the correlation between pre-treatment inflammatory biomarkers and the post-operative depth of invasion (DOI) and worst pattern of invasion (WPOI) in early-stage oral tongue squamous cell carcinoma (OTSCC) by means of positive sentinel lymph node biopsy (SLNB). A retrospective analysis of patients affected by cN0 T1-T2 OTSCC who had undergone an SLNB at the National Cancer Institute of Naples was performed. The patients were studied using an evaluation of the pre-treatment neutrophil-to-lymphocyte ratio (NLR), platelet-to-lymphocyte ratio (PLR), systemic immune-inflammatory index (SII), and a histopathological analysis of the DOI and WPOI. The statistical analysis showed that among the prognostic biomarkers, the NLR was a significant predictor of high WPOI values (*p* = 0.002). The cut-off NLR value was 2.52 with a probability of developing a positive sentinel lymph node biopsy (SLNB) of 30.3%. In contrast, the DOI value was 5.20 with a probability of developing a positive SLNB of 31.82%. Regarding the WPOI, increasing the WPOI class increased the likelihood of a positive SLNB occurrence, and a positive significant correlation was found between the WPOI and SLNB (C_sp_ = 0.342; *p* < 0.001). Pre-treatment NLR, together with post-surgical DOI and WPOI, can be a reliable predictor of occult neck metastasis in patients affected by early-stage OTSCC with a clinically negative neck. Further prospective studies with a larger series will be needed to confirm the results obtained and to better define the NLR, WPOI and DOI cut-off values in order for elective neck dissection to be recommended in relation to a clinically negative neck.

## 1. Introduction

Oral squamous cell carcinoma (OSCC) is the most common malignancy of the oral cavity and the tongue is its most common subsite (oral tongue squamous cell carcinoma, OTSCC) [1]. Due to the rich lymphatic network of the oral cavity, cervical lymph node metastasis can easily occur. In the literature, there is no reservation in asserting the usefulness of elective neck dissection for T3-T4 cN0 OTSCC. However, for T1-T2 cN0 OTSCC, there are several suggested therapeutic strategies, including sentinel lymph node biopsy (SLNB), elective neck dissection and watchful waiting, the latter being the least frequently practiced [2,3].

SLNB is a reliable method for the detection of cervical metastasis in cN0 T1-T2 OTSCC when the primary tumor can be reliably excised with adequate security margins [4]. Using peritumor infiltration with a radioactive tracer, it is possible to identify the lymph nodes that first drain the tumor area so that they can be selectively removed. In 2015, the Sentinel European Node Trial (SENT), a large multicenter trial which involved 14 centers around Europe, reported a total sensitivity and a negative predictive value in the controlled national bank of 86% and 95%, respectively, in a sample of 415 patients [5].

Elective neck dissection, which involves the removal of the lymph node stations closer to the tumor site, is the most widely used approach in international literature. It guarantees greater oncological safety with regard to the control of occult metastases, but it is not free from complications. In fact, this surgical treatment of the neck can lead to post-operative complications in a variable percentage of cases, ranging from 20 to 40% [2].

Tsushima et al. [6] highlighted the low predictive ability in the definition of current pre-treatment markers of occult cervical metastases in stage I and II squamous cell carcinoma of the oral tongue. For this reason, the search for new parameters capable of identifying patients who are at a higher risk of relapse would seem to be mandatory in order to avoid overtreatment of the neck and to ensure prognostic success.

The most recent (8th) American Joint Committee on Cancer (AJCC) TNM classification includes the depth of invasion (DOI) of the primary tumor as a staging parameter which must be taken into account by the surgeon in the therapeutic planning [7]. The DOI is defined as the distance from the deepest level of invasion to the mucosal surface [7]. The DOI is the most reliable prognostic parameter because patients presenting a high-tumor DOI (usually >4 mm) are more likely to present lymph node metastasis [8,9,10]. Histological predictive models have been widely studied for OSCC and the worst pattern of invasion (WPOI) was a consistent component of these models [11]. In particular, the WPOI is the highest score of the pattern of invasion (POI) present in the surgical specimen and represents a pathological parameter related to the DOI value in OSCC [12]. The POI is the worst manner of infiltration present at the tumor/host interface and includes five types of patterns with increasing negative prognostic value: broad pushing tumor front (POI 1); finger-like pushing invasion (POI 2); large tumor islands > 15 cells (POI 3); small tumor islands ≤ 15 cells or tumor strands (POI 4); and satellite tumor nodules at least 1 mm away from the main tumor (POI 5) [11]. Furthermore, the literature has clearly established the relationship between biomarkers of systemic inflammation and oncogenesis and the former’s role as negative prognostic factors in tumors of various districts [13]. In head and neck oncology, the pre-treatment neutrophil-to-lymphocyte ratio (NLR) is a biomarker of particular importance due to its capacity to predict the risk of occult neck metastasis in early-stage OTSCC [14]. The NLR is determined in blood samples by dividing the number of neutrophils by the number of lymphocytes. It is a marker of inflammation and a well-known negative prognostic factor in oncology [13,14]. 

This study aimed to investigate the correlation between the pre-treatment inflammatory biomarkers and the post-operative DOI and WPOI in early-stage OTSCC by means of positive sentinel lymph node biopsy. The authors have aimed to obtain additional information which can guide the surgeon in selecting the most appropriate treatment strategy regarding the neck in cN0 T1-T2 OTSCC.

## 2. Materials and Methods

A retrospective analysis of patients affected by cN0 T1-T2 OTSCC who had undergone an SLNB, treated between January 2006 and January 2021 at the Maxillo-Facial and Ear Nose and Throat (ENT) Surgery Department of the National Cancer Institute (Istituto Nazionale Tumori, Naples, Italy) “IRCCS Pascale” of Naples, was performed. Since 2005, the National Cancer Institute IRCCS Pascale of Naples has been a centre of reference for SLNB of the head and neck district. The data were retrieved from the institutions database. This study was conducted in accordance with the Helsinki Declaration, and due to its retrospective nature, the approval of the local ethics board was not required. Written informed consent was obtained from all the patients before any diagnostic or therapeutic procedure, relating also to the publication of their data in an anonymous form.

The inclusion criteria were as follows:

- a histologically-proven case of early-stage OTSCC (T1-T2);
- OTSCC with no regional or distant metastasis detectable at presentation, clinically and radiographically (neck ultrasound, CT, and/or MRI) (cN0-cM0);
- treatment with excision of the primary tongue tumor and SLNB;
- primary OTSCC that had not been previously treated;
- no clinical history of radiotherapy or chemotherapy treatment;
- no previous cancers at any other sites;
- a WPOI evaluation on a post-operative specimen using previously published guidelines: WPOI 1: pushing border, WPOI 2: finger-like growth, WPOI 3: large islands, >15 cells each; WPOI 4: small islands, ≤15 cells each; WPOI 5: satellites ≥1 mm away from the main mass or other satellites;
- no clinical conditions that might affect the neutrophil-to-lymphocyte ratio (NLR), platelet-to-lymphocyte ratio (PLR), and systemic immune-inflammatory index (SII), (infection, autoimmune hematological disease, history of corticosteroid therapy or chronic renal insufficiency).

The diagnostic workflow for all the patients included: a clinical examination, a routine blood sample with a liver and renal function test, a pre-operative flexible fibro-pharyngoscopy, a neck ultrasound with doppler, head and neck CT or MRI with contrast, a lymphoscintigraphy to identify the sentinel lymph node and a histopathological examination on the incisional biopsy. For all patients, clinical evaluation was performed by a head and neck surgeon, which included a clinical assessment of the tumor site and an evaluation of the diagnostic images and biopsy result in order to evaluate the surgical strategy. To obtain an updated histological description, all the OTSCCs were retrospectively classified according to the 8th AJCC classification, and all the post-operative specimens analyzed before 2019, stored for scientific purposes in dedicated laboratories, were retrospectively evaluated in order to also consider the WPOI value. The pre-operative NLR was obtained using the absolute neutrophil count divided by the absolute lymphocyte count, while the PLR was obtained using the ratio between the platelet count and absolute lymphocyte count. The systemic immune-inflammatory index (SII) was calculated as the (neutrophil count) × (platelet count)/(lymphocyte count). 

### Statistical Analysis

Descriptive statistics are reported as mean (standard deviation (SD)) or median [interquartile range (IQR)]. A non-parametric Kruskal–Wallis test was performed to identify statistically significant differences in the DOI, NLR, PLR and SII versus the WPOI, considering all the included patients and all the patients with a positive SLNB. Linear regression modeling was used to evaluate the most significant predictor and best linear combination of the DOI, NLR, PLR and SII versus the WPOI. A logistic regression model was used to estimate the probability *π*(*x*) of a positive SLNB by studying the NLR and DOI. The analysis was performed using Newton–Raphson’s iterative algorithm.

$$\pi \left(x\right)=\frac{\exp\left(intercept+\beta x\right)}{1+\exp\left(intercept+\beta x\right)}$$

The procedure used was based on the maximum likelihood estimation (MLE) of the parameters in accordance with Newton–Raphson’s iterative algorithm. The same logistic regression model was used to study the relationship between the DOI and a positive SLNB. The Pearson correlation coefficient was calculated with respect to the DOI, PLR, NLR and SII. The Spearman correlation coefficient was calculated with respect to the WPOI and SLNB.

The chi-squared test was used to identify statistically significant differences in prevalence between groups.

Statistical analysis was performed using MATLAB R2007a Statistics Toolbox (MathWorks, Natick, MA, USA) and a *p* value < 0.05 was considered significant.

## 3. Results

A total of 137 patients were treated for early-stage OTSCC at our institute within the review period. Of these cases, a total of 31 patients were excluded because they did not fulfil the inclusion criteria. In particular, 15 patients who had undergone surgery before 2018 were excluded from the study because of a DOI > 10 mm, which according to the 8th AJCC classification, corresponds to a T3; six patients were excluded because of a history of radio/chemotherapy; five patients were excluded due to a clinical condition affecting the NLR and five patients were excluded because they were already suffering from a neoplasm at another site. Consequently, there were 106 patients who met the inclusion criteria and were therefore involved in the study. The mean age of the general population was 65.25 (range 24–93), and the M:F sex ratio was about 1:1 (51M:55F). Half of the patients were smokers (55/106), 18% (19/106) reported a daily alcohol consumption, and 9% (10/106) reported both a smoking and drinking habit. The SLNB positivity was 33% (35/106). The neck lymph nodes level most frequently associated with the SLNB was level II (64%, 68/106), followed by level III (34%, 30/106) and level I (8%, 9/106). Levels IV and V were investigated in four cases and one case, respectively. The most frequently recorded tumor grading was G2 (50%, 53/106), followed by G3 (32%, 34/106), while G2-G3 and G1 were both recorded in around 9% of cases. Neuronal and vascular invasion were both observed in 15% of cases (16/106), and a combination of these two conditions was recorded in five cases. The mean DOI value was 5.36 mm, with a range between 0.5 and 9.5 mm. WPOI III and II were the most frequently recorded scores in 30% of cases (32/106) and 27% of cases (29/106), respectively, followed by WPOI IV (21%, 23/106), WPOI V (12%, 13/106) and WPOI I (8%, 9/106). The NLR mean value was 2.89, with a range between 0.96 and 13.42. The PLR mean value was 160.3, with a range between 25.6 and 562.3. The SII mean value was 693.5 with a range between 76.8 and 3365.12. Among patients with a positive SLNB, the mean age was in line with the general population (64, range 33–91), with a slight female predominance observed (M:F 1:1.3). The habits trend was superimposable with that of the general population. A positive SLNB was recorded principally in patients at level II (23/35, 66%), with a positivity index of 33% (23/68), followed by level III with 25% (9/35) and a positivity index of 30%, (9/30). Level I, IV, and V, recorded just one positive biopsy. G3 was the most frequently observed tumor grading (62% of cases, 22/35), followed by G2 in 31% of cases, (11/35). Only two cases of G2–G3 were documented. Vascular invasion and neuronal invasion were recorded in 28 and 22% of cases, respectively, with two cases reporting both conditions. The DOI mean value was 5.65 mm, with a range between 0.9 and 9.5 mm. WPOI IV was the most frequently recorded pattern (37% of cases, 13/35), followed by WPOI V (22%, 8/35), WPOI III (20%, 7/35), WPOI II (14%, 5/35), and WPOI I (5%, 2/35). The NLR mean value was 3.5, with a range between 1.31 and 13.42, the PLR mean value was 176.1, with a range between 88.18 and 562.3 and the SII mean value was 888.9, with a range between 286 and 3365.1 (Table 1).

The Mann–Whitney U test was used to assess the significance of median differences in the NLR, PLR and SII between patients with positive and negative SLNB. These differences were significant for the NLR (SLNB- 2.35 [IQR 1.86–3.11] versus SLNB+ 2.77 [IQR 2.21–3.86]; *p* = 0.037), but not for the PLR (SLNB− 138 [IQR 107.8–192.14] versus SLNB+ 144.73 [IQR 123.18–217.3]; *p* = 0.219) and the SII (SLNB− 552.24 [IQR 388.87–753] versus SLNB+ 681.2 [IQR 448–1037.4]; *p* = 0.07).

In the Kruskal–Wallis test, a statistically significant difference was observed in the entire population in terms of the DOI and NLR (Figure S1) compared to the WPOI class.

Considering the NLR and SII values in the group of patients with a positive SNLB, the results showed a statistically significant difference compared to the WPOI class (Figure S2). The best linear regression model in terms of identifying the WPOI class showed that the most significant predictors were the DOI and NLR (Table 2) with respect to all patients.

The best linear regression model in terms of identifying the WPOI class showed that the most significant predictor was the NLR (Table 3) in patients with a positive SLNB.

The mathematical relationship between the NLR and the probability of developing a positive SLNB was obtained with a logistic regression model using the equation in (2) which produced the following parameters:

Intercept_NLR = −1.6

β_NLR = 0.3045

**Figure 1:**
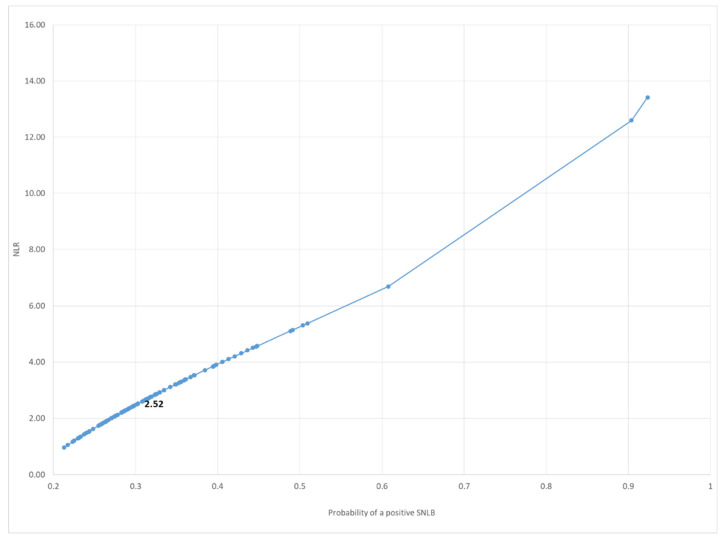
Logistic model: the mathematical relationship between the NLR and the probability of developing a positive SLNB.

The mathematical relationship between the NLR and the probability of developing a positive SLNB was analysed as a curve (Figure 1). The corresponding NLR value was 2.52 with a probability of developing a positive SLNB of 30.3%.

The mathematical relationship between the DOI and the probability of developing a positive SLNB was obtained with a logistic regression model using the equation in (3) which produced the following parameters:

Intercept_DOI = −1.066

β_DOI = 0.06476

**Figure 2:**
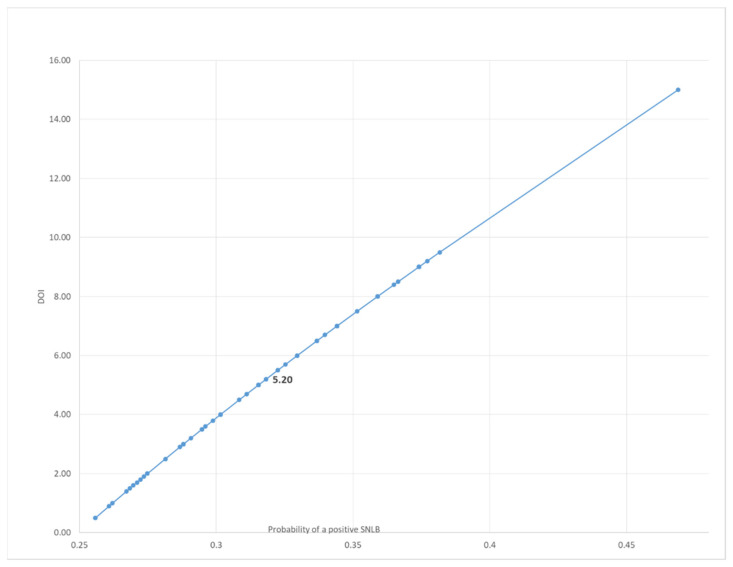
Logistic model: the mathematical relationship between the DOI and the probability of developing a positive SLNB.

The mathematical relationship between the DOI and the probability of developing a positive SLNB was analysed as a curve (Figure 2). The corresponding DOI value was 5.20 with a probability of developing a positive SLNB of 31.82%.

**Figure 3:**
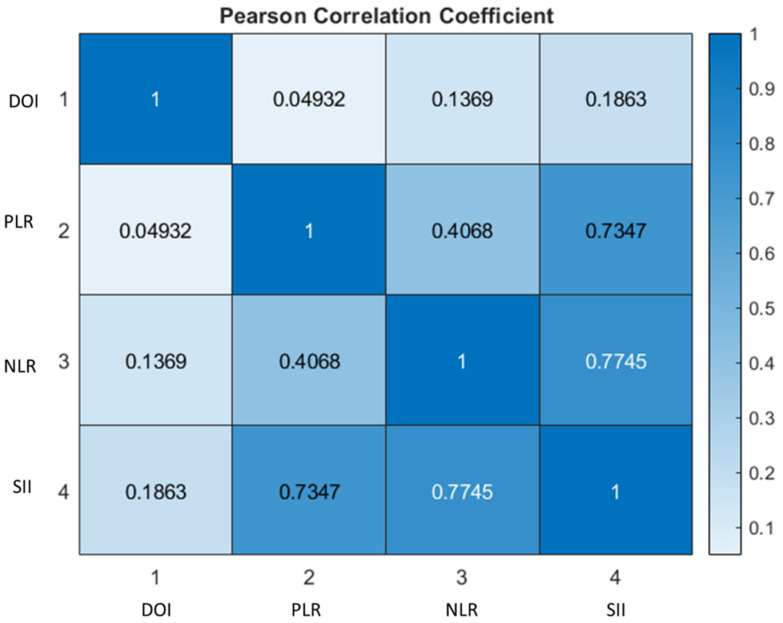
Heat map of the Pearson correlation coefficient between the DOI, PLR, NLR and SII.

Figure 3 reports that a good correlation was obtained between the PLR and SII (0.74) and between the NLR and SII (0.77).

Table 4 reports the distribution of the WPOI between patients with a positive and negative SLNB.

Increasing the WPOI class increased the likelihood of the occurrence of a positive SLNB (*p* = 0.003 at the Chi square test). A significant positive correlation was found between the WPOI and SLNB (C_Sp_ = 0.342; *p* < 0.001).

These statistical analyses show that among the prognostic biomarkers, the NLR was a significant predictor of high WPOI values (*p* = 0.002). The cut-off NLR value was 2.52 with a probability of developing a positive sentinel lymph node biopsy (SLNB) of 30.3%. In contrast, the DOI value was 5.20 with a probability of developing a positive SLNB of 31.82%. Regarding the WPOI, the increase in the WPOI class increased the likelihood of a positive SLNB. Considering the values from WPOI III, the probability of lymph node metastasis was greater than the established cutoff of 20%.

## 4. Discussion

Cervical lymph node metastasis is one of the most important negative prognostic factors in OTSCC and almost 25% of patients with OTSCC show lymph node metastases at presentation [15,16]. In the case of nodal metastasis, the survival rates decrease by as much as 50% with respect to N1 cancer [17].

The 8th and most recent AJCC TNM classification includes the DOI as a staging parameter capable of helping the surgeon in the surgical planning and excludes extrinsic muscle invasion (EMI) from the T categorization [18]. The DOI can be measured post-operatively on the surgical specimen to determine the pathological TNM (pTNM) on the pre-operative imaging by drawing a perpendicular line passing through the basal membrane of the normal squamous epithelium up to the deepest tumor invasion point, obtaining the clinical TNM (cTNM) or on the biopsy specimen, obtaining the biopsy TNM (bTNM) [8,19]. The DOI of the primary tumor is considered to be one of the most reliable prognostic parameters and patients presenting a high-tumor DOI are more likely to present lymph node metastasis, which represents an indication for elective neck dissection [9]. Huang et al. reported a DOI greater than 4 mm as a strong predictor of cervical lymph node involvement [20]. Salzano et al. found that a DOI measurement of 5.43 mm was associated with a 50% probability of developing occult neck metastases, which was not yet evident clinically or by using current imaging techniques [14].

Several studies have considered obtaining a value for the tumor thickness pre-operatively, applying imaging techniques such as magnetic resonance (MRI) and ultrasound [21]. Rocchetti et al. [22] evaluated the relationship between the pre-treatment tongue ultrasound and the surgical specimen DOI, describing a moderate correlation. The use of ultrasound also helped to distinguish between two patterns of growth pre-operatively, a vertical and a horizontal pattern, with the worse prognosis in the former, due to higher and faster infiltration. Multiparameter histological predictive models have been widely studied in relation to OSCC, with the tumor pattern of invasion (POI) being a consistent component of these models [11,23]. An important post-operative pathological parameter, evaluated on the surgical specimen, is the WPOI; whereas, the pattern of invasion has been identified as a criterion for tumor invasiveness in OSCC by several authors, such as Bryne et al. and Brandwein-Gensler et al. [24,25,26]. The WPOI refers to the worst pattern of infiltration present in the specimen at the tumor/host interface, no matter how focal [27]. The WPOI in cN0 early-stage OTSCC has been identified as a valuable predictor of disease-free survival [12]. Puy et al. analyzed the relationship between the WPOI and DOI in relation to OSCC and demonstrated that the DOI can vary according to the POI, and that patients with WPOI I-III had a low DOI value and those with WPOI IV-V a high DOI value [20]. Marinelli et al. revealed an association between the WPOI and DOI in OSCC, with infiltrative tumors showing more aggressive invasive patterns, and reported that patients with WPOI V were 32 times more likely to die of the disease than patients with WPOI I-IV, with WPOI V being considered equivalent to perineural invasion [28]. The literature shows that the WPOI is usually evaluated on the surgical specimen post-operatively, but as suggested by Seki M et al., the WPOI can also be studied on a biopsy specimen. This approach can be considered effective in predicting the prognosis in cN0 early-stage OSCC and could guide surgical planning [29].

Nowadays, the main strategy for the management of patients with early-stage OTSCC is still a matter of debate [30,31,32]. The significant number of available and quite comparable treatment options leads to the need to determine new factors in order to assess the best surgical solution. The identification of additional markers associated with an increased risk of occult metastasis in early-stage OTSCC supports the choice of the most appropriate neck treatment strategy [33]. The role of inflammation in tumor oncogenesis has already been established and hematological markers of inflammation have emerged as prognostic factors for several cancers, influencing survival outcomes [34]. The NLR, PLR and SII are biomarkers of systemic inflammation which can be evaluated in a pre-operative peripheral blood sample [35,36]. In the literature, the use of pre-treatment hematological biomarkers, such as the NLR and PLR, as prognostic factors for several cancers has been described [13]. The negative prognostic value of these biomarkers has already been highlighted in the literature in relation to tumors of the head and neck district. Abbate et al. demonstrated a statistically significant relationship between a pre-operative NLR > 2.93 and the risk of neck occult metastasis in patients with OTSCC [37]. Salzano et al. found that the pre-operative NLR and DOI values in OTSCC are linearly associated with a positive correlation: a unit increase (1 mm) in the DOI corresponds to an 0.47 increase in the NLR. Moreover, a pre-treatment NLR of 2.93 and a DOI value of 5.43 mm are associated with a 50% probability of developing occult neck metastasis [14].

To the best of our knowledge, this is the first study to evaluate the correlation between high pre-operative levels of the NLR and a high post-operative WPOI in relation to positive SLNB early-stage cN0 OTSCC.

In our population, a higher mean value of the NLR was observed in the SLNB+ group, associated with a greater incidence of high WPOI values compared to the SLNB- group. The statistical analysis shows that, among the prognostic biomarkers, the NLR was the most significant predictor of high WPOI values (*p* = 0.002). The PLR and SII revealed a mild correlation, the SII, in particular, showed a good correlation with the NLR. The pre-treatment NLR was also identified as a predictor of the probability of developing a positive SLNB, a value of 2.52 corresponding to a 30.3% probability of developing an occult metastasis. This finding was supported by differences in the NLR in SLNB+ and SLNB- patients which proved significant using the Mann–Whitney U test. The prognostic role of the NLR is already well known in the literature and this is probably due to the different subtypes of inflammatory response and cytokine activation, which are important promotors of tumor growth [37]. A comparable likelihood of developing a positive SLNB was observed when studying the post-operative DOI value, 5.20 mm corresponding to a 31% probability of presenting occult cervical metastases. For both values, the increase in probability is linear, as illustrated in Figure 1 and Figure 2. In agreement with the literature, both values of the NLR and DOI indicate elective neck dissection as the recommended surgical treatment since they are higher than the established value of 20% for the risk of developing neck metastasis.

Furthermore, we have also demonstrated that increasing the WPOI class increases the likelihood of the occurrence of a positive SLNB (*p* = 0.002). A positive significant correlation was found between the WPOI and SLNB (C_Sp_ = 0.342; *p* < 0.001). Despite the patients having been screened for the evaluation of inflammatory pathologies which could involve the values of the NLR, PLR and SII, these values are subject to changes linked to factors that are not foreseeable. Therefore, this circumstance could have altered the data and represents one of the limitations of the study, together with its retrospective nature.

## 5. Conclusions

The choice of the strategy for the management of patients with early-stage OTSCC with a cN0 neck remains a much-debated topic in the literature. Successively, many authors have highlighted the necessity of additional prognostic factors to consider during the decisional progress. Prognostic factors, inflammatory biomarkers, the DOI and WPOI have progressively gained a fundamental role in the stratification of patients with OTSCC, offering the possibility of predicting the probability of neck metastasis and therefore of selecting the most appropriated therapeutic strategy. Considering the results of our study, while accepting that there are only a small number of cases, we would like to recommend the use of the NLR, WPOI and DOI as additional tools for the prediction of the occurrence of occult neck metastasis and therefore for the planning of an elective neck dissection in early-stage OTSCC (cT1-T2).

We would not suggest performing an SLNB in the case of a high pre-treatment NLR, but in such circumstances, would advise waiting for the post-surgical DOI and WPOI values of the OTSCC. If, on the basis of these parameters, the probability of developing occult metastasis seems to be greater than 20%, we would suggest performing an elective neck dissection once the post-operative histological examination has been obtained. Moreover, patients with a high WPOI score in the incisional biopsy could be candidates for elective neck dissection concomitant with tumor excision.

Further prospective studies on a larger series will be needed to confirm the results obtained and to better define the NLR, WPOI and DOI cut-off values appropriate for the recommendation of an elective neck dissection in relation to a clinically negative neck.

## Figures and Tables

**Table 1:** Characteristics of the SLNB + population. Legend: + yes, − no.

Gender	Age	Smoking	Drinking	Level	Type and Grade	Vascular Invasion	Neural Invasion	DOI	WPOI	N	L	P	NLR	PLR	SII
M	45	−	−	III	SCC, G2	−	−	5.2 mm	II	2.9	2.2	271	1.31	123.18	355.01
M	42	−	−	II	SCC, G2	−	−	6 mm	I	2.3	1.7	222	1.35	130.5	299.7
M	46	−	−	III	SCC, G3	−	−	3.2 mm	III	2.6	1.7	194	1.52	114.11	196.7
F	79	−	−	II	SCC, G2	−	−	4 mm	III	3.1	1.7	225	1.82	132.35	409.5
M	56	−	−	II	SCC, G3	+	−	5 mm	II	3.4	1.8	251	1.88	139.44	474.1
F	48	+	−	II	SCC, G3	+	−	4 mm	II	4.2	2.2	272	1.9	123.6	516.8
F	81	−	−	II	SCC, G2	−	−	3.8 mm	I	1.8	0.9	143	2	158.8	286
F	86	−	−	III	SCC, G3	+	−	9 mm	III	3.3	1.5	148	2.2	98.66	325.6
M	84	+	−	III	SCC; G2–G3	−	−	0.9 mm	II	4.2	1.9	200	2.21	105.26	442
F	71	+	−	II	SCC, G3	−	+	8 mm	III	3	1.3	233	2.3	179.2	542.9
F	53	+	−	II	SCC, G2	−	−	7 mm	IV	3.5	1.5	192	2.33	128	448
M	80	+	−	II	SCC, G2–G3	+	−	3.5 mm	IV	5.3	2.2	194	2.4	88.18	467.36
M	70	−	−	III	SCC, G3	+	+	8.5 mm	V	5.4	2.2	199	2.45	90.45	487.55
F	65	−	−	I	SCC, G3	−	−	1.6 mm	III	4.8	1.9	253	2.52	133.15	637.56
F	70	−	−	V	SCC, G3	−	−	3.6 mm	IV	7.3	2.8	262	2.6	93.57	681.2
M	54	+	−	III	SCC, G2	−	−	4 mm	II	7.5	2.8	306	2.67	109.28	819.6
F	42	−	−	II	SCC, G3	+	+	6 mm	III	3.3	1.2	381	2.75	317.5	1047.75
F	65	−	+	III	SCC, G3	−	−	2 mm	V	2.5	0.9	137	2.77	152.22	379.49
M	74	+	−	III	SCC, G2	−	−	2.9 mm	IV	3.8	1.3	208	2.92	160	607.36
F	81	−	−	II	SCC, G3	−	−	6 mm	IV	4.8	1.6	338	3	211.25	1014
F	68	+	−	II	SCC, G3	−	−	8 mm	IV	5.7	1.9	275	3	144.73	825
M	55	+	−	II	SCC, G3	−	+	8 mm	V	4.5	1.4	233	3.21	166.42	747.93
M	70	+	−	II	SCC, G3	−	+	8.5 mm	IV	5.4	1.6	171	3.37	106.87	577.12
F	72	+	−	II	SCC, G3	−	+	8 mm	V	4.4	1.3	212	3.38	163.07	717.58
F	79	−	−	II	SCC, G2	−	+	8.4 mm	IV	5.2	1.5	326	3.46	217.3	1127.96
F	62	+	−	II	SCC, G3	−	−	9 mm	V	6	1.7	956	3.52	562.3	3365.12
F	71	+	−	II	SCC, G3	+	−	4 mm	IV	5.8	1.5	251	3.86	167.33	970.53
M	91	+	+	III	SCC, G3	+	−	7 mm	III	4.3	1.1	322	3.9	292.72	1258.7
F	42	+	−	II	SCC, G3	−	−	5 mm	IV	3.6	0.9	255	4	283.33	1020
M	77	+	−	IV	SCC, G2	+	−	1.7 mm	IV	8.2	2	278	4.1	139	1139.80
F	53	+	−	II	SCC, G3	+	−	8 mm	IV	4.1	0.9	228	4.55	253.33	1037.40
F	74	−	+	II	SCC, G2	−	−	5 mm	V	3.6	0.7	179	5.14	255.71	920.06
M	61	−	−	II	SCC, G3	−	−	8 mm	V	8.7	1.3	198	6.69	123.75	1324.60
M	33	+	+	II	SCC, G2	−	+	10 mm	IV	12.6	1	259	12.6	259	3263.40
F	52	−	−	II	SCC, G3	−	−	5 mm	V	9.4	0.7	170	13.42	242.85	2281.40

**Table 2:** Linear regression model considering the DOI, PLR, NRL and SII with respect to the WPOI class in all patients.

Linear Regression Model	Coefficients	*p*-Value
Intercept	1.786626656	4.29488 × 10^−6^
DOI	0.173377001	4.78043 × 10^−5^
PLR	−0.001428808	0.50874393
NLR	0.130388283	0.018422181
SII	0.000190121	0.690418221

**Table 3:** Linear regression model considering the DOI, PLR, NRL and SII with respect to the WPOI class in the patients with a positive SLNB.

Linear Regression Model	Coefficients	*p*-Value
Intercept	2.074314876	0.002333873
DOI	0.115348849	0.149478113
PLR	0.001657338	0.652856978
NLR	0.179003139	0.016464027
SII	−0.00010145	0.879207812

**Table 4:** Distribution of the WPOI between patients with a positive and negative SLNB.

WPOI	Positive SLNB	Negative SLNB	Probability	95% CI	*p*-Value at Chi Square Test
1	2	7	10.2%	3.9–23.8	0.003
2	5	24	18.3%	10.3–30.5	
3	7	25	30.7%	22.1–40.9	
4	13	10	46.6%	34.2–59.4	
5	8	5	63.2%	43.2–79.5	

## Data Availability

G.S. and F.I. had full access to all of the data in the study and take responsibility for the integrity of the data and the accuracy of the data analysis.

## Supplementary Materials

The following supporting information can be downloaded at: https://www.mdpi.com/article/10.3390/jpm12111931/s1, Figure S1: S1a Box plot of the DOI with respect to the WPOI class. S1b Box plot of the NLR with respect to the WPOI class. S1c Box plot of the PLR with respect to the WPOI class. S1d Box plot of the SII with respect to the WPOI class. Figure S2: S1a Box plot of the DOI in all the patients with a positive SLNB with respect to the WPOI class. S1b Box plot of the NLR in all the patients with a positive SLNB with respect to the WPOI class. S1c Box plot of the PLR in all the patients with a positive SLNB with respect to the WPOI class. S1d Box plot of the SII in all the patients with a positive SLNB with respect to the WPOI class.
