# Supplementary material for: Early-Stage Oral Tongue Squamous Cell Carcinoma and a Positive Sentinel Lymph Node Biopsy: Description of a Prognostic Correlation between Pre-Treatment Inflammatory Biomarkers, the Depth of Invasion and the Worst Pattern of Invasion
Source: J Pers Med. 2022 Nov 19;12(11):1931. doi: 10.3390/jpm12111931 (PMC9692700; doi:10.3390/jpm12111931)
Supplement: Supplementary file 1 [file jpm-12-01931-s001.zip › jpm-1949951-supplementary.pdf]

Journal of
*Personalized
Medicine*

MDPI

SUPPLEMENTARY FIGURES

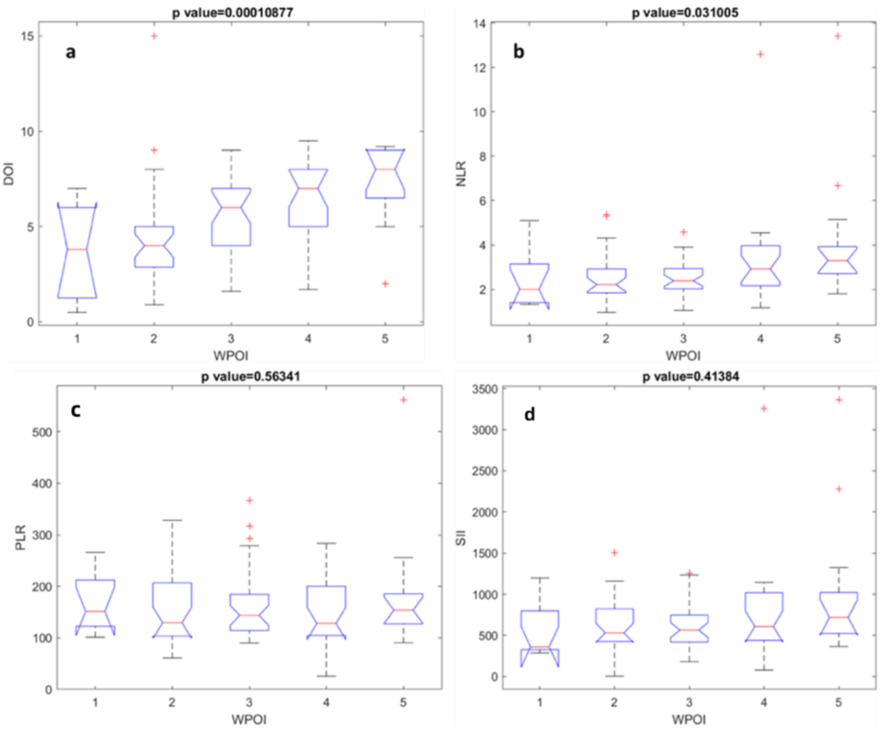

Figure S1. Box plots of the DOI, NLR, PLR and SII in all the patients with respect to the WPOI class. S1a Box plot of the DOI with respect to the WPOI class. S1b Box plot of the NLR with respect to the WPOI class. S1c Box plot of the PLR with respect to the WPOI class. S1d Box plot of the SII with respect to the WPOI class.

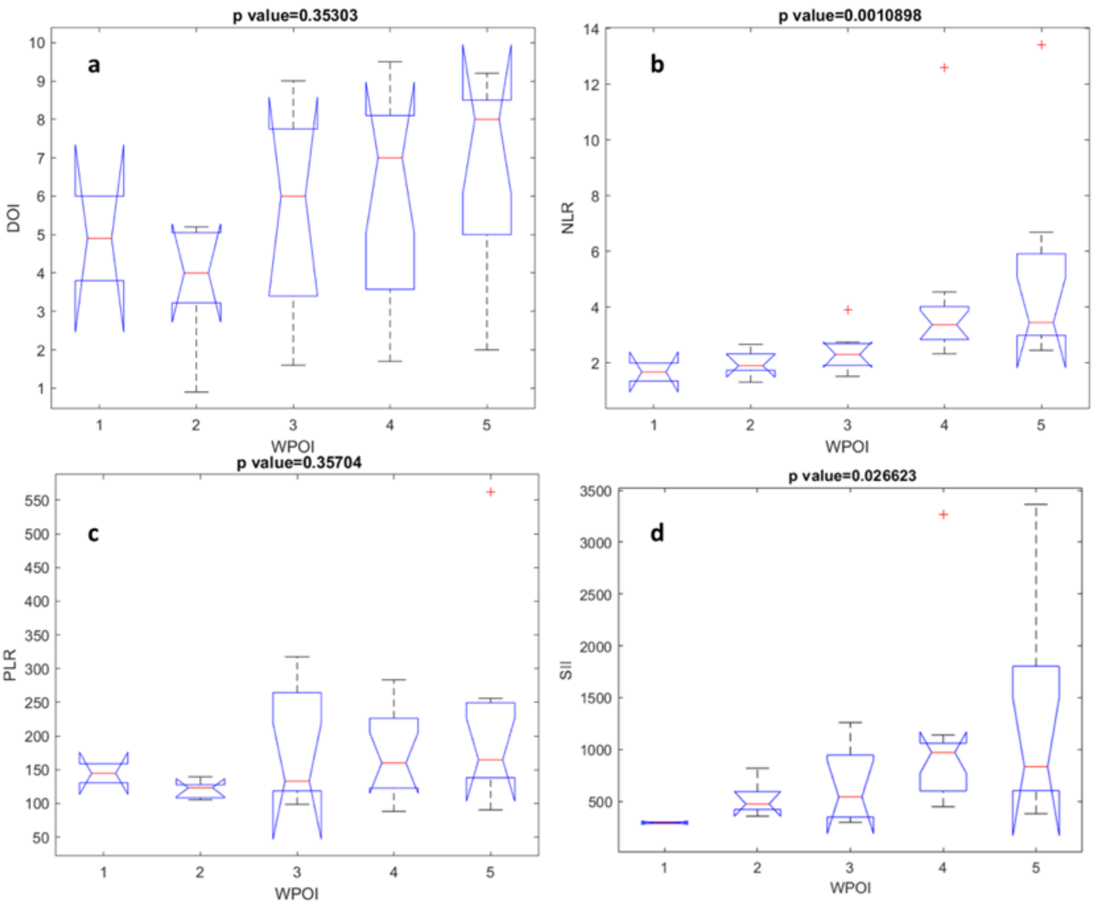

Figure S2. Box plots of the DOI, NLR, PLR and SII in all the patients with a positive SLNB with respect to the WPOI class. S1a Box plot of the DOI in all the patients with a positive SLNB with respect to the WPOI class. S1b Box plot of the NLR in all the patients with a positive SLNB with respect to the WPOI class. S1c Box plot of the PLR in all the patients with a positive SLNB with respect to the WPOI class. S1d Box plot of the SII in all the patients with a positive SLNB with respect to the WPOI class.
